# Alkali-Metal-Mediated Manganation(II) of Functionalized Arenes and Applications of *ortho*-Manganated Products in Pd-Catalyzed Cross-Coupling Reactions with Iodobenzene

**DOI:** 10.1002/chem.200701597

**Published:** 2007-11-21

**Authors:** Victoria L Blair, William Clegg, Ben Conway, Eva Hevia, Alan Kennedy, Jan Klett, Robert E Mulvey, Luca Russo

**Affiliations:** [a] WestCHEM, Department of Pure and Applied Chemistry, University of StrathclydeGlasgow, G1 1XL, UK; [b] School of Natural Sciences (Chemistry), Newcastle UniversityNewcastle upon Tyne, NE1 7RU, UK

**Keywords:** aromatic compounds, cross-coupling, manganese, metalation, sodium

## Abstract

Extending the recently introduced concept of “alkali-metal-mediated manganation” to functionalised arenes, the heteroleptic sodium manganate reagent [(tmeda)Na(tmp)(R)Mn(tmp)] (**1**; TMEDA = *N*,*N*,*N*′,*N*′-tetra-methylethylenediamine, TMP = 2,2,6,6-tetramethylpiperidide, R = CH_2_ SiMe_3_) has been treated with anisole or *N*,*N*-diisopropylbenzamide in a 1:1 stoichiometry in hexane. These reactions afforded the crystalline products [(tmeda)Na(tmp)(*o*-C_6_H_4_OMe)Mn(tmp)] (**2**) and [(tmeda)Na(tmp){*o*-{C(O)N(*i*Pr)_2_C_6_H_4_}Mn(CH_2_SiMe_3_)] (**3**), respectively, as determined from X-ray crystallographic studies. On the basis of these products, it can be surmised that reagent **1** has acted, at least partially and ultimately, as an alkyl base in the first reaction liberating the silane Me_4_Si, but as an amido base in the second reaction liberating the amine TMPH. Both of these paramagnetic products **2** and **3** have contacted ion-pair structures, the key features of which are six-atom, five-element (NaNMnCCO) and seven-atom, five-element (NaNMnCCCO) rings, respectively. Manganates **2** and **3** were successfully cross-coupled with iodobenzene under [PdCl_2_(dppf)] (dppf=1,1′-bis(diphenylphosphino)ferrocene) catalysis to generate unsymmetrical biaryl compounds in yields of 98.0 and 66.2%, respectively. Emphasizing the importance of alkali-metal mediation in these manganation reactions, the bisalkyl Mn reagent on its own fails to metalate the said benzamide, but instead produces the monomeric, donor–acceptor complex [Mn(R)_2_{(*i*Pr)_2_NC(Ph)(=O)}_2_] (**5**), which has also been crystallographically characterised. During one attempt to repeat the synthesis of **2**, the butoxide-contaminated complex [{(tmeda)Na(R)(OBu)(*o*-C_6_H_4_OMe)Mn}_2_] (**6**) was obtained. In contrast to **2** and **3**, due to reduced steric constraints, this complex adopts a dimeric arrangement in the crystal, the centrepiece of which is a twelve atom (NaOCCMnC)_2_ ring.

## Introduction

Recently we added manganese(II) to the growing list of unlikely metals that can perform *direct* metalation of aromatic compounds, when driven by alkali-metal mediation.^[1]^ Such metalations (metal–hydrogen exchange reactions) are usually the domain of highly reactive, highly polar organometallics, most typically alkyllithium or lithium amide compounds.^[2]^ Previously, to bind Mn^II^ to a carbon atom of an aromatic compound would normally require a prelithiation step, followed by a salt metathesis (commonly with a Mn^II^ halide).^[3]^ However, despite its widespread applicability, this two-step approach is not all-conquering as lithiation suffers from limited functional group tolerance and low kinetic stability, while the ionicity of metal halide salts can lead to solubility problems in common organic solvents. On their own, organomanganese(II) complexes, as comparatively low-polarity organometallics, are generally too weakly basic to effect C-H deprotonation at a reasonable rate, but when paired with an alkali metal in a heterobimetallic ate, they can transform into super-manganating reagents. This alkali-metal-mediated manganation (AMMMn) and alkali-metal-mediated metalation (magnesiation, zincation, alumination)^[4]^ in general can potentially circumvent the obstacles stated above, but they are profoundly more than direct alternative methods to indirect metathetical manganations (magnesiations etc.), being synergic metalations capable of delivering unprecedented products and structures inaccessible by means of metathesis. The initial examples of AMMMn demonstrate this emphatically, producing in the case of benzene, the tetrasodium dimanganese amido inverse crown with a benzenediide core [Na_4_Mn_2_(tmp)_6_(C_6_H_4_)],^[5]^ (TMP=2,2,6,6-tetramethylpiperidide) and with ferrocene, the dilithium dimanganese trinuclear ferrocenophane [(tmeda)_2_Li_2_Mn_2_{Fe(C_5_H_4_)_2_}_3_] (TMEDA=*N*,*N*,*N*′,*N*′-tetramethylethylenediamine).^[1]^ Describing here the first study in which AMMMn has been applied to functionalised arenes, we report the surprising finding that the synergic heteroleptic base employed reacts differently, seemingly depending on the directing ability (in a directed *ortho*-metalation (DoM) sense) of the substituent group on the aryl ring. Furthermore, we reveal the structures of these sodium arylmanganated intermediates, the first structures of their type, and show how they can be successfully cross-coupled with iodobenzene under palladium catalysis to generate unsymmetrical biaryls.

## Results and Discussion

**Scheme 1:**
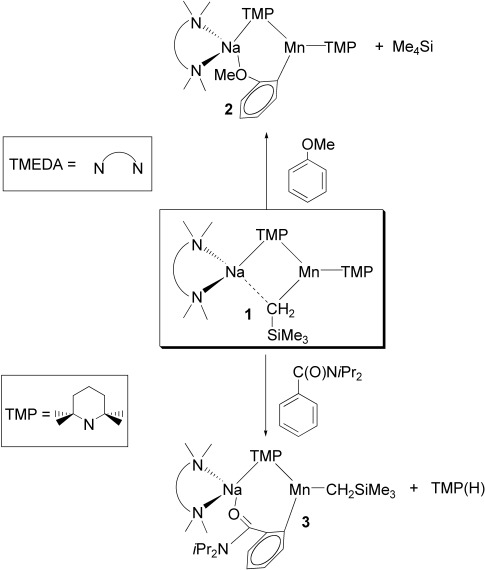
Ambibasic behaviour of reagent 1 towards anisole and *N*,*N*-diisopropylbenzamide affording the *ortho*-manganated products 2 and 3, respectively.

The sodium monoalkyl–bisamidomanganate [(tmeda)Na(tmp)(R)Mn(tmp)] (**1**; R=CH_2_SiMe_3_) was used as the AMMMn reagent in the new reactions. Only one previous reaction of **1** has been reported,^[5]^ namely with benzene, in which it acted as an alkyl (R) base to produce the phenyl derivative [(tmeda)Na(tmp)(Ph)Mn(tmp)] and tetramethyl-silane. Here, prepared in situ in hexane, **1** was treated with a molar equivalent of the appropriate functionalised arene (anisole or *N*, *N*-diisopropylbenzamide; Scheme 1) selected on the basis of their respective contrasting weak and strong DoM ability.^[6]^ Both reactions afforded a crystalline product in yellowish-green [(tmeda)Na(tmp)(*o*-C_6_H_4_OMe)Mn(tmp)] (**2**) and orange [(tmeda)Na(tmp){*o*-[C(O)N(*i*Pr)_2_]C_6_H_4_}Mn(R)] (**3**), respectively. Isolated yields were 66 and 31%, respectively.

In both **2** and **3**, the arene molecules have been selectively *ortho*-deprotonated, with a Mn^II^ atom occupying the vacated hydrogen site (see structures below); thus, to the best of our knowledge these syntheses represent the first examples of directed *ortho*-manganations of substituted arenes.^[7]^ However, there is a major distinction between **2** and **3**. Following the benzene precedent, **1** operates ultimately as an alkyl base towards anisole, generating tetramethylsilane and **2**, which consequently has a monoaryl–bisamido composition. In contrast, **1** exhibits amido basicity towards the tertiary amide, leading to the monoalkyl–monoaryl–monoamido **3** with concomitant elimination of TMPH. This unexpected result establishes that **1** is a versatile manganating reagent with a basicity switch (potentially tuneable to alkyl or amido) seemingly dependent on the nature of the aromatic substrate. Clearly distinct mechanisms must therefore be available to **1** in its reactions with anisole and *N*,*N*-diisopropylbenzamide. Of course, at this stage all we can definitely conclude from structurally characterizing the products from these reactions is that manganating reagent **1** functions *ultimately* as an alkyl base towards anisole and as an amido base towards benzamide. Thermodynamically the loss of volatile Me_4_Si from such manganations is likely to be greatly preferred. However, as recently discussed in the context of alkyl–TMP–zincates,^[8]^ there may be an additional intermediate step in which TMP acts firstly as the base, but then re-enters the coordination sphere of the alkali metal as TMP(H) ligand, before reacting with the alkyl ligand to eliminate alkane and to restore the TMP anion to the heterobimetallic structure. Thus the distinction between **2** and **3** may be attributed to kinetic factors. Intriguingly, subjecting the same tertiary amide to the related zincate base [(tmeda)Na(tmp)(*t*Bu)Zn(*t*Bu)], induces *ortho*-zincation ultimately not through amido (TMP) basicity, but through alkyl (*t*Bu) basicity.^[9]^ Thus the basicity switch in these synergic metalations is also dependent on the identity of the σ-bonding divalent metal (and coligand set) within the base, as changing these parameters will also change the kinetic profile of the metalation reaction.

**Figure 1:**
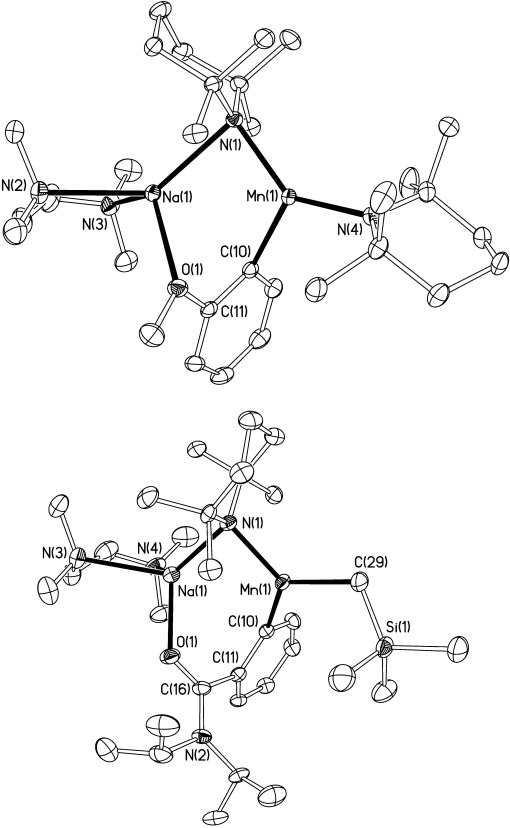
Molecular structures of 2 (top) and 3 (bottom) with 30% probability displacement ellipsoids. H atoms have been omitted for clarity. In addition for 3, minor disorder components have been omitted for clarity, and only one of two crystallographically independent molecules is shown.

Due to the paramagnetic nature of **2** and **3**, NMR spectroscopy could not be used for their characterisation. We therefore turned to X-ray crystallography. The molecular structures of **2** and **3** (Figure 1 and Table 1) share common general features. Classifiable as contacted ion pairs, both structures display a trigonal planar Mn coordination (N,N,C in **2**; C,N,C in **3**) comprising one terminal (TMP in **2**, R in **3**) and two bridging ligands (TMP in both; *o*-deprotonated anisole and benzamide in **2** and **3**, respectively). Defining the metalations as manganations, the deprotonated *ortho*-C atoms form σ bonds with the Mn atoms, but do not interact with the Na atoms. Instead the aryl ligands bridge to Na through their *O*-heteroatoms to close six-membered (NaNMnCCO) and seven-membered (NaNMnCCCO) rings in **2** and **3**, respectively, with bidentate TMEDA ligands completing the distorted tetrahedral (N,N,N,O) environments of Na. A search of the Cambridge Structural Database^[10]^ revealed **2** and **3** to be unique with no hits found for ether- or amide-substituted aryls with *ortho*-Mn^II^ atoms^[11]^ or indeed in the latter case with any *ortho*-transition-metal atoms. Furthermore alkali-metal-arylmanganated structures of any type are surprisingly rare with no sodium examples at all (besides those previously made by AMMMn^[1^,^5]^). Power has reported^[12]^ three lithium examples, significantly all synthesised from metathesis reactions, and only one, [Li(thf)_4_]-[MnMes_3_], has trigonal planar Mn, but in a very different (cf. **2** and **3**) solvent-separated ion-pair arrangement. Unfortunately, disorder within **3** limits the precision of its metrical parameters, though the connectivities are definite, and the main features of its two independent molecules are similar to those of **2**. The best comparison with **2** is provided by the unsubstituted phenyl analogue [(tmeda)Na(tmp)(Ph)Mn(tmp)]^[5]^ (**4**). Lack of aryl substitution gives **4** a smaller four-membered (NaNMnC) ring with a modestly longer Mn-C bond (2.207(4) Å to *ipso*-C cf. 2.189(2) Å to *ortho*-C in **2**) made by the Mn atom lying almost coplanar with the aryl ring plane (deviation 0.339 Å cf. 0.558 Å in **2**; 0.221 and 0.220 Å for the two independent molecules of **3**). With respect to the MnN_2_C planes, the aryl rings have dihedral angles of exactly 90° by symmetry in **4** and 71.4° in **2**. A long Na-*ipso*-C bond in **4** (2.731(4) Å) is replaced by a significantly shorter Na-O(Me) bond in **2** (2.5357(16) Å), reflecting the greater bond strength of donor–acceptor dative interactions versus cation–π interactions;^[13]^ the Na-O bonds in **3** are even shorter, at 2.313(6) and 2.309(5) Å.

**Table 1:** Key bond lengths [Å] and angles [°] for **2**,**3**, **5** and **6**.

	**2**	**3**[c]	**5**	**6**
Mn-N (TMP)	2.0930(17)^a^ 2.0298(17)^b^	2.102(4)^a^ 2.093(4)^a^	–	–
Mn-C (R)	–	2.158(5)^b^ 2.159(6)^b^	2.159(2) 2.165(2)	2.177(2)
Mn-C (Aryl)	2.189(2)	2.171(5) 2.172(5)	–	2.177(2)
Mn-O	–	–	2.1724(14) 2.1415(15)	2.1333(15) 2.1318(16)
Na-N (TMP)	2.5387(19)	2.493(5) 2.495(5)	–	–
Na-O	2.5357(16)	2.313(6) 2.309(5)	–	2.3997(18) 2.2982(17)^d^
Na-C (R)	–	–	–	3.047(3)
N-Mn-N	133.36(6)	–	–	–
N-Mn-C (R)	–	122.8(2)	–	–
N-Mn-C (Aryl)	111.19(7)^a^ 115.44(7)^b^	115.08(18)	–	–
C-Mn-C	–	120.7(2)	127.43(11)	126.08(9)
O-Mn-O	–	–	96.96(6)	84.42(6)
O-Mn-C (R)	–	–	111.73(9) 103.76(8) 102.66(8) 110.26(8)	110.10(8) 102.37(7)
O-Mn-C (Aryl)	–	–	–	110.05(7) 115.92(7)

[a] Bridging.

[b] Terminal.

[c] Two molecules in asymmetric unit.

[d] O-*n*Bu; R=CH_2_SiMe_3_

**Scheme 2:**
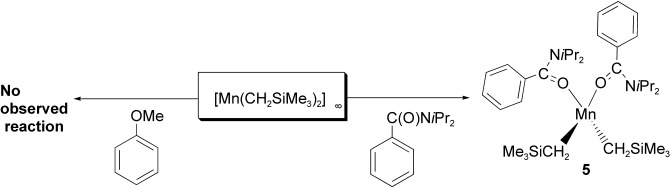
Contrasting reactivity of [Mn(CH_2_SiMe_3_)_2_]_∞_ towards anisole and *N*,*N-*diisopropylbenzamide.

**Figure 2:**
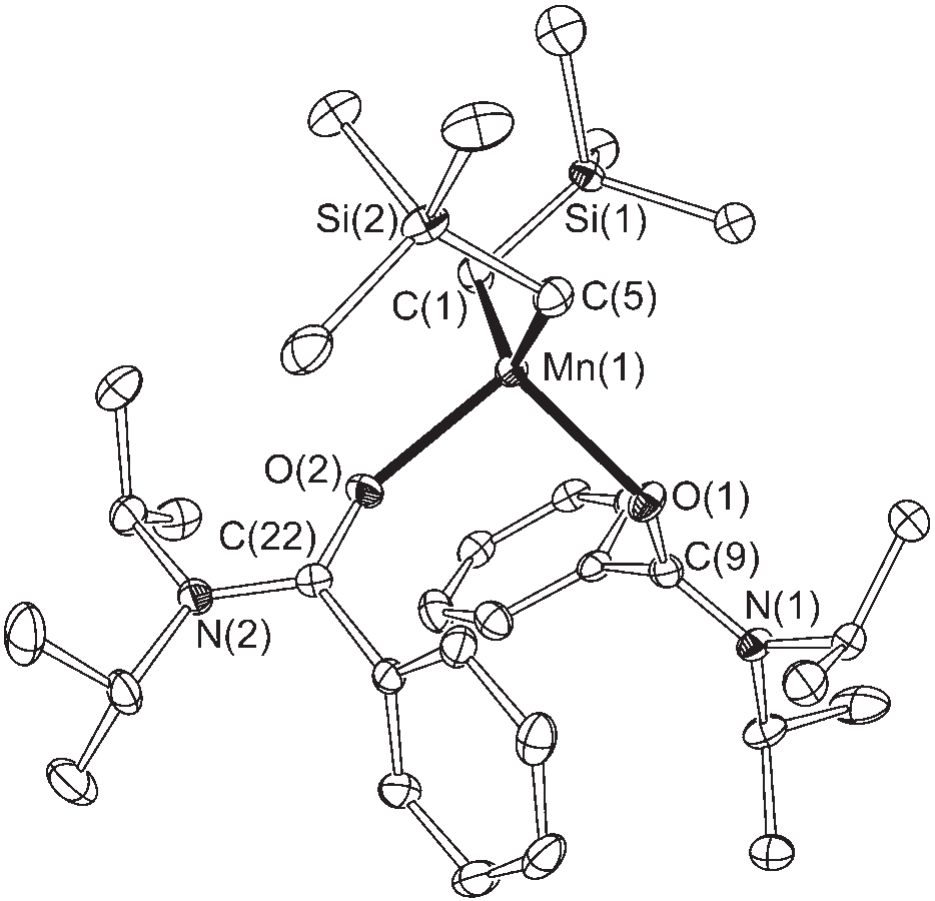
Molecular structure of 5 with 30% probability displacement ellipsoids. H atoms and minor disordering have been omitted for clarity.

It is worth emphasising that the organomanganese reagent Mn(CH_2_SiMe_3_)_2_ on its own failed to metalate either anisole or *N*,*N-*diisopropylbenzamide and did not act as a nucleophile towards the latter. Thus, no reaction was observed at all when the bisalkyl reagent was mixed with anisole, whereas the reaction with *N*,*N-*diisopropylbenzamide afforded the Lewis acid/Lewis base association complex [Mn(CH_2_SiMe_3_)_2_{(*i*Pr)NC(Ph)(=O)}_2_] (**5**) as orange crystals in a 24% yield, which could be improved to 63% when the reaction was carried out with two molar equivalents of the benzamide (Scheme 2). Determined by X-ray crystallography, the molecular structure of **5** (Figure 2 and Table 1) has a Mn atom in a distorted tetrahedral environment made up of two alkyl groups and two neutral benzamide ligands, which coordinate to the metal through their oxygen atoms. To the best of our knowledge the only precedent for a crystallographically characterised homometallic^[14]^ compound containing a neutral tertiary aromatic amide coordinated to a metal is the tris(alkyl) gallium complex [*t*Bu_3_Ga{O=C(Ph)NMe_2_}].^[15]^ The Mn-C bond lengths in **5** (2.159(2), 2.165(2) Å) are nearly identical to those found in the related monomeric complex [Mn(CH_2_SiMe_3_)_2_{(−)-sparteine}]^[16]^ (2.1582(18), 2.165(17) Å). In addition, the Mn-O bond length (2.1724(14), 2.1415(15) Å) are similar to that in the THF adduct of the related manganese bis(alkyl) species [Mn{CH(SiMe_3_)_2_}_2_(thf)] (2.19(2) Å),^[17]^ although in the latter the Mn centre is tricoordinate due to the heavier silylation of the ligand, whereas in **5** there are four ligands around the metal. The structure of the unsolvated manganese compound is known to be a polymer,^[18]^ thus the formation of **5** illustrates the superior Lewis basicity of *N*,*N*-diisopropylbenzamide relative to that of anisole, which cannot accomplish the same cleavage of polymeric [Mn(CH_2_SiMe_3_)_2_]_∞_. Cocomplexation of the bis(alkyl)manganese reagent, or fragment thereof, with an alkali metal amide leads to a discrete molecular structure as seen with **1**, hence the problem of having to cleave a polymeric structure is not an issue. This is one advantageous factor of the mixed-metal synergy inherent in reagents such as **1**.

**Scheme 3:**
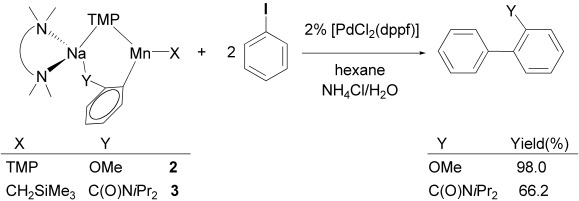
Palladium-catalysed cross-coupling reactions of 2 and 3 with iodobenzene.

To the best of our knowledge alkali metal arylmanganates have not been previously studied in the context of transition-metal-catalysed cross-coupling reactions with aryl halides. Precedents exist for organomanganese(II) halides of general formula ArMnX in excellent work reported by Cahiez.^[19]^ Since **2** and **3** have covalent Mn-C (aryl) bonds within anionic ate-activated structures, we reasoned they could make excellent coupling agents as playing only a secondary, Lewis acidic role in the structures, sodium should not be detrimental even though ionic arylsodium reagents are not generally suitable for coupling applications. Test reactions with iodobenzene in hexane in the presence of 2% [PdCl_2_(dppf)] (Scheme 3; dppf=1,1′-bis(diphenylphosphino)ferrocene) confirmed this analysis. In the case of **2**, the coupled product 2-methoxybiphenyl was obtained in an impressive isolated yield of 98.0%. Coupling was also successful with **3**, though the yield of isolated *N*,*N*-diisopropyl-2-phenylbenzamide was lower at 66.2%. Interestingly when the experiment with **2** was repeated in the absence of the Pd catalyst direct coupling was still observed albeit in a reduced yield (32.0%). The same catalyst-free experiment with **3** was even more successful with a less reduced yield of 47%.

**Scheme 4:**
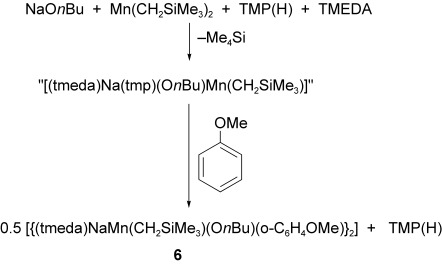
Possible reaction pathway for the formation of butoxide-containing 6.

High purity in the reagents employed and complete exclusion of oxygen in the system are crucially important for the success of these metalation applications. Thus, when the reaction that afforded **2** was repeated with a sample of *n*-butylsodium contaminated by *n*-butoxide, compound [{(tmeda)Na(R)(OBu)(*o*-C_6_H_4_OMe)Mn}_2_] (**6**) was serendipitously obtained (Scheme 4). This new heterotrianionic compound bears a resemblance to **2**, as both contain a molecule of anisole selectively *ortho*-manganated and a [Na(tmeda)]^+^ ion. However they differ in the rest of the anionic ligands supporting the Mn atom. Thus, for **2** two TMP ligands are found, whereas in **6** there is an alkyl and a butoxide anion. A plausible pathway that could account for the synthesis of **6** would involve the initial formation of a heteroleptic manganate species “[(tmeda)Na(tmp)(OBu)Mn(CH_2_SiMe_3_)]” that would react with anisole as an amido base liberating TMPH as coproduct of the reaction. However we must mention that all the attempts to prepare this potentially new mixed-metal reagent have been unsuccessful to date.

**Figure 3:**
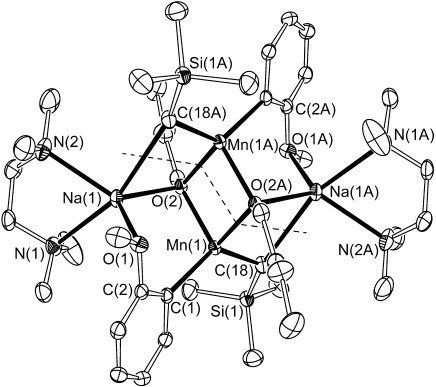
Molecular structure of 6 with 30% probability displacement ellipsoids. H atoms have been omitted for clarity. The dashed line represents the dimerisation junction. Symmetry operator A: −*x*+1, −*y*+1, −*z*.

**Figure 4:**
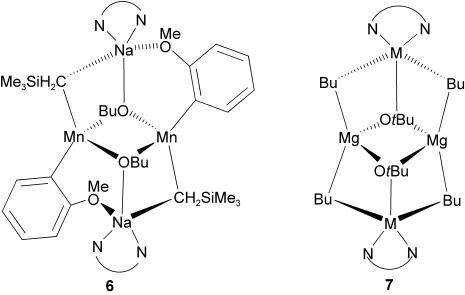
Comparison between the structural motifs of 6 and 7.

The centrosymmetric molecular structure of **6** (Figure 3 and Table 1) was successfully determined by X-ray crystallographic studies. Unlike **2** and **3** which are monomeric structures, **6** comprises two identical bridging cationic {(tmeda)NaMn(OBu)(*o-*C_6_H_4_OMe)(CH_2_SiMe_3_)} units dimerised through alkoxide and alkyl bridges. This structure can be envisaged as a cationic twelve-membered [(NaOCCMnC)_2_]^2+^ ring hosting in its interior two butoxide ligands (Figure 4). This structural motif is reminiscent of that previously found for the series of inverse crown complexes [{M^1^M^2^(N*i*Pr_2_)_2_}_2_X_2_] (M^1^=Li or Na; M^2^=Mg; X=OR, H),^[20]^ [{(tmeda)MMg(Bu)_2_}_2_(O*t*Bu)_2_] (**7**) (M=Na, K)^[21]^ (Figure 4), and [K_2_Ca_2_{OC(=CH_2_)Mes}_6_(thf)_2_] (Mes=mesityl)^[22]^ and for several other homometallic and heterometallic systems.^[4]^ These compounds exhibit a cationic eight-membered ring hosting two anionic ligands. Due to the ambidentate nature of the *ortho*-metalated anisole ligand, C-bonding to Mn and O-bonding to Na, an expansion in ring size from eight atoms in **7** to twelve atoms in **6** is observed.

**Figure 5:**
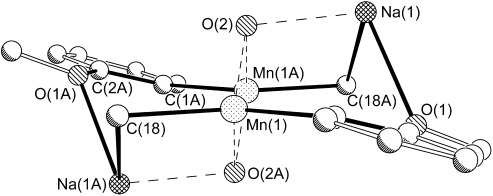
Core of 6 highlighting its pseudo-chair conformation of the [(NaOCCMnC)_2_] ring.

This 12-membered ring adopts a pseudo-chair conformation (Figure 5) with the sodium atoms displaced on either side of the plane defined by C(1)Mn(1)C(18)⋅⋅⋅ C(1A)Mn(1A)C(18A). The oxygen atoms of the anisole groups are also slightly out of this plane as shown by the torsion angle O(1)-C(2)-C(1)-Mn(1), 17.1°. The sodium atoms achieve pentacoordination by bonding to two anionic (C of the alkyl; O of the alkoxide) ligands, the neutral oxygen atom of the metalated anisole, and the two TMEDA nitrogen atoms. There is a slight contraction of the Na-O(*n*Bu) bond length (2.2982(18) Å) relative to that for the OMe group of the anisole ligand (2.3997(18) Å), reflecting the anionic nature of the former even though the latter oygen atom has a lower coordination number. Surrounded by four anionic ligands, the Mn atom has a distorted tetrahedral environment comprising two O*n*Bu, one *ortho*-aryl C and one alkyl C atoms. The Mn-O bond lengths (2.1333(15) and 2.1318(16) Å) are modestly longer than the ones found in the mixed Li/Mn^II^ alkoxides^[23]^ [Li{Mn(N(SiMe_3_)_2_(OC*t*Bu_3_)_2_}] (2.019(4), 1.984(4) Å) and [Li_2_{MnBr_2_(OC*t*Bu_3_)_2_(thf)_2_}] (2.019(7) Å), which can be attributed to the dimeric nature of **6**, in which each butoxide ligand is tri-coordinated, whereas the other structures have two-coordinate alkoxide ligands being monomers. The Mn-C(anisole) (2.177(2) Å) bond length is similar to the one found in **2** (2.189(2) Å).

## Conclusion

In conclusion, the new concept of AMMMn has been successfully applied to arenes functionalised with electron-donating (MeO) or electron-withdrawing {C(O)N(*i*Pr)_2_} groups. Probing the structural changes that accompany the Mn^II^–H exchange reactions revealed that the mixed-metal reagent can function ultimately as an alkyl or amido base depending on the specific arene. When the alkali metal is absent from the solution mixture, the bisalkyl Mn reagent can no longer metalate the benzamide, but instead forms a coordination complex with it. With sodium tamed within a cage of heteroatoms distant from the active carbanion–Mn centre, the bimetallic products of AMMMn can in turn be successfully cross-coupled with iodobenzene to form unsymmetrical biaryls. Overall these results serve to broaden significantly the opportunities for synthesizing new compounds and building new structures in organomanganese(II) chemistry. The sensitivity of these mixed-metal organometallic compounds to air and moisture was demonstrated through the serendipitous synthesis of an *n*-butoxy derivative. The adventitious presence of the *n*BuO anion, which has good bridging capabilities and is not sterically demanding, leads to an increase in aggregation number (monomer to dimer) of the mixed-metal complex and to coordination expansion about the manganese atom (trigonal planar to tetrahedral).

## Experimental Section

All reactions were carried out under a protective argon atmosphere by using standard Schlenk techniques. Hexane was distilled from sodium/benzophenone. ^1^H NMR spectra were recorded on a Bruker DPX 400 MHz spectrometer; data for X-ray crystal structure determination were obtained with a Nonius Kappa CCD and a Bruker SMART 1 K CCD diffractometer using graphite monochromated Mo_K*α*_ radiation (*λ*=0.71073 Å). Selected crystallographic data for compounds **2**, **3**, **5** and **6** are given in Table 2. CCDC 656944 (**2**), 656945 (**3**), 663055 (**5**) and 661429 (**6**) contain the supplementary crystallographic data for this paper. These data can be obtained free of charge from The Cambridge Crystallographic Data Centre via http://www.ccdc.cam.ac.uk/data_request/cif. The IR-spectra were recorded on a Nicolet Avator 360 FT-IR spectrometer and elemental analyses were carried out on a Perkin Elmer 2400 elemental analyser. Melting/decomposition points were measured with a Büchi Melting Point B-545 apparatus.

**Table 2:** Selected crystallographic data for compounds **2**, **3**, **5** and **6**.^a^

	**2**	**3**	**5**	**6**
formula	C_31_H_59_MnN_4_NaO	C_32_H_63_MnN_4_NaOSi	C_34_H_60_MnN_2_O_2_Si_2_	C_42_H_86_Mn_2_N_4_Na_2_O_4_Si_2_
*M_r_* [g mol^−1^]	581.75	625.88	639.96	923.19
crystal system	triclinic	orthorhombic	triclinic	triclinic
space group	*P* 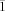	*Pca*2_1_	*P* 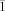	*P* 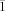
*a* [Å]	10.059(3)	21.854(4)	9.9826(3)	10.949(2)
*b* [Å]	11.212(4)	11.239(3)	10.9135(3)	11.583(3)
*c* [Å]	15.952(5)	31.590(8)	20.2736(6)	12.071(3)
*α* [°]	75.986(5)	90	100.345(2)	72.369(3)
*β* [°]	74.632(5)	90	95.144(2)	67.101(3)
*γ* [°]	87.910(5)	90	113.836(2)	84.003(3)
*V* [Å^3^]	1682.3(9)	7759(3)	1954.91(10)	1343.8(5)
*Z*	2	8	2	1
*ρ*_calcd_ [g cm^−3^]	1.148	1.072	1.087	1.141
*μ*(Mo_Kα_) [mm^−1^]	0.433	0.409	0.427	0.568
measured reflns	11833	17286	39613	7472
independent reflns	5782	8970	8595	4469
observed reflns^b^	5325	7363	5987	3962
parameters	356	1085	405	310
*R*1^c^ (*R*1 all data)	0.039 (0.043)	0.054 (0.068)	0.0469 (0.0861)	0.0378 (0.0442)
*wR*2^d^ (*wR*2 all data)	0.0959 (0.0986)	0.1299 (0.1396)	0.0859 (0.1000)	0.0962 (0.1036)
max/min peaks [e Å^−3^]	+0.84/−0.40	+0.49/−0.29	+0.609/−0.398	+0.55/−0.40

[a] All data were collected at 150 K using Mo_Kα_(*λ*=0.71073 Å) radiation.

[b] Observation criterion: *I*>2σ(*I*).

[c] *R*1=∑||*F*_o_|−|*F*_c_||/∑|*F*_o_|.

[d] *wR*2 = {∑[*w*(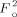
−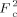)^2^]/∑[w(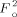)^2^]}^1/2^.

**[(tmeda)Na(tmp)(*o*-C_6_H_4_OMe)Mn(tmp)] (2)**: NaTMP was prepared in situ by reaction of BuNa (0.16 g, 2 mmol) with TMPH (0.34 mL, 2 mmol). Mn(CH_2_SiMe_3_)_2_ (0.46 g, 2 mmol), TMPH (0.34 mL, 2 mmol) and TMEDA (0.30 mL, 2 mmol) were then introduced and the mixture was stirred for 30 min affording a pale yellow solution. At this stage anisole (0.22 mL, 2 mmol) was added and the resulting bright yellow solution was stirred for 18 h at room temperature. The solution was concentrated by removing some solvent under vacuum. Storage of this solution at room temperature afforded a crop of yellow-green crystals after two days (0.76 g, 66%); one of them was employed in an X-ray diffraction experiment. M.p. 128°C (decomp); elemental analysis calcd (%) for C_31_H_59_MnN_4_NaO (581.75): C 64.00, H 10.22, N 9.63; found: C 64.00, H 10.45, N 9.23.

**[(tmeda)Na(tmp){*o*-[C(O)N(*i*Pr)_2_]C_6_H_4_}Mn(R)] (3)**: Following the same experimental procedure as described for **2**, compound **1** was prepared in situ by reaction of BuNa (0.16 g, 2 mmol), TMP(H) (0.68 mL, 4 mmol), Mn(CH_2_SiMe_3_)_2_ (0.46 g, 2 mmol) and TMEDA (0.30 mL, 2 mmol). *N*,*N′*-diisopropylbenzamide (0.41 g, 2 mmol) was then introduced and the mixture was stirred for 18 h affording a bright orange solution that was filtered and concentrated by removing some solvent under vacuum. Storage of this filtrate at room temperature afforded a crop of orange crystals (0.41 g, 31%) after two days; one of them was employed in a X-ray diffraction experiment. M.p. 148°C (decomp); IR (nujol): 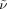 = 1592.7 cm^−^ (C=O); elemental analysis calcd (%) for C_32_H_63_MnN_4_NaOSi (625.88): C 61.41, H 10.15, N 8.95; found: C 61.40, H 10.08, N 8.45.

**Cross-coupling of 2 with iodobenzene**: Iodobenzene (0.44 mL, 4.0 mmol) was added to a crude hexane solution of **2** (2.0 mmol), followed by [PdCl_2_(dppf)] (32.6 mg, 0.04 mmol). After stirring for 18 h under reflux conditions, the mixture was quenched with a saturated NH_4_Cl (5 mL) solution, hexane (15 mL) and distilled water (15 mL). The crude bilayer was filtered through Celite into a separating funnel, with the aqueous layer subsequently discarded. The organic layer was then washed with distilled water (15 mL×3), dried under anhydrous MgSO_4_ for 1 h and then filtered through Celite to produce a clear yellow solution. The solvent was next removed in vacuo and then dissolved in the minimum volume of hexane, which was purified by SiO_2_ column chromatography with pure hexane as the eluant to give 2-methoxybiphenyl (0.36 g, 98.0 %). The same reaction was repeated following the same experimental procedure in the absence of the Pd catalyst, obtaining 2-methoxybiphenyl in a reduced yield (0.12 g, 32%). ^1^H NMR (400 MHz, CDCl_3_, 20°C, TMS): *δ*=7.59 (d, *J*=7.2 Hz, 2H), 7.45 (t, *J*=7.3 Hz, 2H), 7.32–7.41 (m, 3H), 7.07 (t, *J*=7.4 Hz, 1H), 7.02 (d, *J*=8.0 Hz, 1H), 3.88 ppm (s, 3H).

**Cross-coupling of 3 with iodobenzene**: Iodobenzene (0.44 mL, 4.0 mmol) was added to a solution of crude **3** (2.0 mmol) in hexane, followed by [PdCl_2_(dppf)] (32.6 mg,0.04 mmol). After stirring for 18 h under reflux conditions, the mixture was quenched with a saturated NH_4_Cl solution (5 mL), and THF (15 mL) was added to produce a clear organic layer. The organic layer was then washed with distilled water (10 mL×3), dried under anhydrous MgSO_4_ for 1 h and then filtered through Celite to produce a clear brown solution. The solvent was next removed in vacuo and subsequent addition of hexane (10 mL) to the residue produced a suspension, which was treated to standard filtration techniques. The fitrate from this second filtration was purified by SiO_2_ column chromatography with AcOEt/hexane (1:6) as an eluant to give *N*,*N*-diisopropyl-2-phenylbenzamide (0.37 g, 66.0%). The same reaction was repeated following the same experimental procedure in the absence of the Pd catalyst, obtaining *N*,*N*-diisopropyl-2-phenylbenzamide in a reduced yield (0.26 g, 47%).^1^H NMR (400 MHz, CDCl_3_, 20°C, TMS): *δ*=7.55–7.57 (m, 2H), 7.27–7.41 (m, 7H), 3.43 (septet, *J*=6.7 Hz, 1H), 3.22 (septet, *J*=6.8 Hz, 1H), 1.52 (d, *J*=6.8 Hz, 3H), 1.28 (d, *J*=6.9 Hz, 3H), 0.89 (d, *J*=6.7 Hz, 3H), 0.33 ppm (d, *J*=6.7 Hz, 3H).

**[Mn(CH_2_SiMe_3_)_2_{(C(O)N(*i*Pr)_2_)C_6_H_5_}_2_] (5)**: Mn(CH_2_SiMe_3_)_2_ (0.23 g, 1 mmol) was suspended in dry hexane (20 mL). *N*,*N*-diisopropylbenzamide (0.41 g, 2 mmol) was added to give an orange solution which was allowed to stir at room temperature for 5 h. The solution was allowed to stand at room temperature overnight to furnish **5** as a crop of yellow crystals (0.40 g, 63%). M.p. 79.2°C; elemental analysis calcd (%) for C_34_H_60_N_2_O_2_ (639.96): C 63.81, H 9.44, N 4.37; found: C 63.81, H 9.38, N, 4.57.

**[{(tmeda)Na(*n*BuO)Mn(μ-C_6_H_4_-OMe)(CH_2_SiMe_3_)}_2_] (6)**: Following the same methodology described for **2**, the mixed-metal reagent [(tmeda)Na(μ-tmp)(μ-CH_2_SiMe_3_)Mn(tmp)] was prepared in situ by reaction of NaTMP (2 mmol), Mn(CH_2_SiMe_3_) (0.46 g, 2 mmol), TMPH (0.34 mL, 2 mmol) and TMEDA (0.31 mL, 2 mmol) affording a yellow solution. Next, anisole (0.22 mL, 2 mmol) was added and the resulting yellow solution allowed to stir at room temperature for 12 h. The reaction solution was filtered and the yellow filtrate concentrated in vacuo. The solution was left to crystallise overnight at room temperature and a crop of orange/yellow crystals was seen to give **6** (0.46 g, 49.8%). Elemental analysis calcd (%) for C_42_H_86_Mn_2_N_4_Na_2_O_4_Si_2_ (923.19): C 54.64, H 9.38, N 6.07; found: C 54.87, H 9.48, N 5.80.
